# Feature Papers in Optical Sensors 2022

**DOI:** 10.3390/s23073696

**Published:** 2023-04-03

**Authors:** Vittorio M. N. Passaro, Yuliya Semenova, Benjamin L. Miller

**Affiliations:** 1 Dipartimento di Ingegneria Elettrica e dell’Informazione (Department of Electrical and Information Engineering), Politecnico di Bari, Via Edoardo Orabona n. 4, 70125 Bari, Italy; 2 School of Electrical and Electronic Engineering, Photonics Research Centre, Technological University Dublin, Grangegorman, D07 ADY7 Dublin, Ireland; 3 Department of Dermatology, University of Rochester, Rochester, NY 14642, USA

Today, optical sensors are the subject of a very significant number of studies and applications. Many well-established technologies, including free-space optics, integrated photonics, and fiber optics approaches, have been developed in recent decades to fabricate and develop increasingly more efficient optical sensors for applications ranging from industrial control to monitoring the environment, biomedical use, and even as part of the Internet of Things.

The purpose of this Special Issue is to publish a set of significant and original papers where our section’s EBMs discuss key topics in the field, particularly review contributions that demonstrate the advancement of optical sensing technology and successfully present new and consolidated application areas. Areas of interest have included the evaluation of new sensors, new sensing principles, new applications, and new technologies, as well as review papers on the state of the art of well-established technologies for sensing. In this Special Issue, fifteen papers of great interest are collected, ranging from fiber Bragg gratings for sensing applications, to laser microfabrication approaches, as well as visible, infrared and Raman spectroscopy.

In the first paper, by Chapalo et al. [1], the authors investigated the gamma radiation response of fiber Bragg gratings (FBGs) inscribed in a few-mode polymer optical fiber. The fiber had a graded-index CYTOP core of 20 µm and XYLEX overclad of 250 µm in diameter. Four FBGs were exposed to gamma radiation during four irradiation sessions at a 5.3 kGy/h dose rate. The FBGs showed a linear Bragg wavelength shift with the received dose with a mean sensitivity of −3.95 pm/kGy at 43 °C. The increased temperature provides a rise in the sensitivity: it reached −10.6 pm/kGy at 58 °C. After irradiation, the FBGs showed partial recovery, which increased with the received dose. Furthermore, the FBG’s reflection power decreased with the dose. This attenuation is mainly due to insertion losses caused by the radiation-induced attenuation in the CYTOP fiber. A linear response to the received dose makes CYTOP FBGs attractive for gamma radiation dosimetry.

In the second paper, by Hong et al. [2], the authors propose a modified two-axis surface encoder to separately measure both the in-plane displacement and the *Z*-directional out-of-plane displacement with minor crosstalk errors. The surface encoder is composed of a scale grating and a small-sized sensor head. In the modified surface encoder, the measurement laser beam from the sensor head is designed to be projected onto the scale grating at a right angle. For measurement of the *X*- and *Y*-directional in-plane scale displacement, the positive and negative first-order diffracted beams from the scale grating are superimposed on each other in the sensor head, producing interference signals. On the other hand, the *Z*-directional out-of-plane scale displacement is measured based on the principle of a Michelson-type interferometer. To avoid the influence of reflection from the middle area of the transparent grating, which causes periodic crosstalk errors in the previous research, a specially fabricated transparent grating with a hole in the middle is employed in the newly designed optical system. A prototype sensor head has been constructed, and basic performances of the modified surface encoder were tested through experiments.

In the third paper, by Yu et al. [3], the authors propose a hybrid laser microfabrication approach for the manufacture of three-dimensional (3D) optofluidic spot-size converters in fused silica glass through a combination of femtosecond (fs) laser microfabrication and carbon dioxide laser irradiation. Spatially shaped fs laser-assisted chemical etching was first performed to form 3D hollow microchannels in glass, which were composed of embedded straight channels, tapered channels, and vertical channels connected to the glass surface. Then, carbon dioxide laser-induced thermal reflow was carried out for the internal polishing of the whole microchannels and sealing parts of the vertical channels. Finally, 3D optofluidic spot-size converters (SSC) were formed by filling a liquid-core waveguide solution into laser-polished microchannels. With a fabricated SSC structure, the mode spot size of the optofluidic waveguide was expanded from ~8 μm to ~23 μm with a conversion efficiency of ~84.1%. Further measurement of the waveguide-to-waveguide coupling devices in the glass showed that the total insertion loss of two symmetric SSC structures through two ~50 μm-diameter coupling ports was ~6.73 dB at 1310 nm, which was only approximately half that of non-SSC structures with diameters of ~9 μm at the same coupling distance. The proposed approach shows great potential for developing novel 3D fluid-based photonic devices for mode conversion, optical manipulation, and lab-on-a-chip sensing.

In the fourth paper, by Xiong et al. [4], the authors propose a new method based on the interferometric pseudo-lateral-shearing method to evaluate the pitch variation of a large-scale planar variable-line-spacing (VLS) grating. In the method, wavefronts of the first-order diffracted beams from a planar VLS grating are measured using a commercial Fizeau form interferometer. Utilizing the differential wavefront of the first-order diffracted beam before and after the small lateral shift of the VLS grating, the pitch variation of the VLS grating can be evaluated. Meanwhile, additional positioning errors of the grating in the lateral shifting process could degrade the measurement accuracy of the pitch variation. To address the issue, the technique referred to as the reference plane technique is also introduced, where the least squares planes in the wavefronts of the first-order diffracted beams are employed to reduce the influences of the additional positioning errors of the VLS grating. The proposed method can also reduce the influence of the out-of-flatness of the reference flat in the Fizeau interferometer by taking the difference between the measured positive and negative diffracted wavefronts (namely, self-calibration can be accomplished). After the theoretical analysis and simulations, experiments were carried out with a large-scale VLS grating to verify the feasibility of the proposed methods. Furthermore, the evaluated VLS parameters were verified by comparing them with the readout signal of an absolute surface encoder employing the evaluated VLS grating as the scale for measurement.

In the fifth paper, by Hou et al. [5], a porcine model was used to investigate the feasibility of using VIS-NIR spectroscopy to differentiate between degrees of ischemia–reperfusion injury in the small intestine. Ten pigs were used in this study and four segments were created in the small intestine of each pig: (1) control; (2) full arterial and venous mesenteric occlusion for 8 h; (3) arterial and venous mesenteric occlusion for 2 h followed by reperfusion for 6 h; and (4) arterial and venous mesenteric occlusion for 4 h followed by reperfusion for 4 h. Two models were built using partial least squares discriminant analysis. The first model was able to differentiate between the control, ischemic, and intestinal segments with an average accuracy of 99.2% with 10-fold cross-validation, and the second model was able to discriminate between the viable versus non-viable intestinal segments with an average accuracy of 96.0% using 10-fold cross-validation. Moreover, histopathology was used to investigate the borderline between viable and non-viable intestinal segments. The VIS-NIR spectroscopy method together with a PLS-DA model showed promising results and appears to be well-suited as a potentially real-time intraoperative, easy-to-use and non-invasive method.

In the sixth paper, by Hsu et al. [6], the authors present the first demonstration of a 3D VLIP system utilizing a two-stage neural network (TSNN) model, where the received-intensity-selective-enhancement scheme, known as RISE, can alleviate the light non-overlap zones in the VLIP system. In a practical test-room with dimensions of 200 × 150 × 300 cm^3^, the experimental results showed that the mean errors in the training and testing data sets are reduced by 54.1% and 27.9% when using the TSNN model with RISE in the *z*-direction, and they are reduced by 39.1% and 37.8% in the *xy*-direction, respectively, when comparing that with using a one stage NN model only. At the cumulative distribution function (CDF) P90, the TSNN model with RISE can reduce the errors by 36.78% when compared with that in the one stage NN model.

In the seventh paper, by Wang et al. [7], a novel micron-range displacement sensor based on a whispering-gallery mode (WGM) microcapillary resonator filled with a nematic liquid crystal (LC) and a magnetic nanoparticle-coated fiber half-taper has been proposed and experimentally demonstrated, where the tip of a fiber half-taper coated with a thin layer of magnetic nanoparticles (MNPs) moves inside the LC-filled microcapillary resonator along its axis. The input end of the fiber half-taper is connected to a pump laser source and due to the thermo-optic effect within the MNPs, the fiber tip acts as point heat source increasing the temperature of the LC material in its vicinity. An increase in the LC temperature leads to a decrease in its effective refractive index, which in turn causes spectral shift of the WGM resonances monitored in the transmission spectrum of the coupling fiber. The spectral shift of the WGMs is proportional to the displacement of the MNP-coated tip with respect to the microcapillary’s light coupling point. The sensor’s operation has been simulated considering heat transfer in the microcapillary filled with a LC material with a negative thermo-optic coefficient. The simulations are in a good agreement with the WGMs spectral shift observed experimentally. A sensitivity to displacement of 15.44 pm/µm and a response time of 260 ms were demonstrated for the proposed sensor. The device also shows good reversibility and repeatability of response. The proposed micro-displacement sensor should have potential applications in micro-manufacturing, precision measurement and medical instruments.

In the eighth paper, by Park et al. [8], the authors introduced a digital photo image analysis in color space to estimate the spectrum of fluor components dissolved in a liquid scintillator sample through the hue and wavelength relationship. Complementary metal oxide semiconductor (CMOS) image sensors with Bayer color filter array (CFA) technology in the digital camera were used to reconstruct and decode color images. Hue and wavelength are closely related. To date, no literature has reported the hue and wavelength relationship measurements, especially for blue or close to the UV region. The non-linear hue and wavelength relationship in the blue region was investigated using a light emitting diode source. They focused on this wavelength region, because the maximum quantum efficiency of the bi-alkali photomultiplier tube (PMT) is around 430 nm. It is necessary to have a good understanding of this wavelength region in PMT-based experiments. The CMOS Bayer CFA approach was sufficient to estimate the fluor emission spectrum in the liquid scintillator sample without using an expensive spectrophotometer.

In the ninth paper, by Riesen et al. [9], the authors have demonstrated an integrated optofluidic sensor consisting of a pillar array-based open microfluidic chip and caged dye-doped whispering gallery mode microspheres, which shows potential for the simple real-time monitoring of liquids. The open microfluidic chip allows for the wicking of a thin film of liquid across an open surface with subsequent evaporation-driven flow enabling a continuous passive flow for sampling. The active dye-doped whispering gallery mode microspheres is placed between pillars, avoiding the use of cumbersome fibre tapers to couple light to the resonators as is required for passive microspheres. The performance of this integrated sensor is demonstrated using glucose solutions (0.05–0.3 g/mL) and the sensor response is shown to be dynamic and reversible. The sensor achieves a refractive index sensitivity of ~40 nm/RIU, with Q-factors of ~5 × 10^3^, indicating a detection limit of ~3 × 10^−3^ RIU (~20 mg/mL glucose). Further enhancement of the detection limit should be expected by increasing the microsphere Q-factor using high-index materials for the resonators, or alternatively, inducing lasing. The integrated sensors are well expected to have significant potential for a host of downstream applications, particularly relating to point-of-care diagnostics.

In the tenth paper, by Calvagna et al. [10], an innovative probe for thermally controlled portable Raman spectroscopy (exc. 785 nm) equipped with infrared sensing lines was developed. It includes an infrared source and two thermopile sensors, allowing one to perform real-time measurements of the local emissivity of the material surface under laser excitation. The emissivity, which is needed in order to monitor the temperature of the irradiated surface through infrared radiation measurements, represents the complementary component of the reflectance in the radiative energy balance. Thus, total reflectance, temperature measurements and Raman spectroscopy were integrated in the probe. After independently assessing the reliability of the former in order to derive the emissivity of variety of materials, the probe was successfully applied on pigments, paint layers, and a painting on canvas. The results provided evidence of the significant exploitation potential of the novel tool.

In the eleventh invited paper, by Liang et al. [11], an in-plane single-quartz-enhanced dual spectroscopy (IP-SQEDS)-based trace gas sensor was demonstrated for the first time. A single quartz tuning fork (QTF) was employed to combine in-plane quartz-enhanced photoacoustic spectroscopy (IP-QEPAS) with light-induced thermoelastic spectroscopy (LITES) techniques. Water vapor (H_2_O) was chosen as the target gas. Compared to traditional QEPAS, IP-SQEDS not only enabled simple structures, but also obtained a signal amplitude enhancement nearly three times higher.

In the twelfth paper, by Barrios [12], a low-cost, smartphone-based optical diffraction grating refractometer is reported. Its principle of operation is based on the dependence of the diffraction efficiency of a DVD grating on the surrounding refractive index. The studied configuration uses the built-in LED flashlight and camera of a smartphone as a light source and a detector, respectively, to image the DVD grating diffraction pattern. No additional optical accessories, such as lenses, fibers, filters, or pinholes, are employed. The refractive index sensor exhibits a linear response in the refractive index range of 1.333–1.358 RIU (refractive index unit), with a sensitivity of 32.4 RIU^−1^ and a resolution of 2 × 10^−3^ RIU at the refractive index of water. This performance makes the proposed scheme suitable for affinity-based biosensing and a promising optosensing refractometric platform for point-of-need applications.

In the thirteenth paper, by Wahrendorff et al. [13], metalworking fluids (MWFs) are widely used to cool and lubricate metal workpieces during processing to reduce heat and friction. Extending a MWF’s service life is of importance from both economical and ecological points of view. Knowledge about the effects of processing conditions on the aging behavior and reliable analytical procedures are required to properly characterize the aging phenomena. In this paper, the authors present a simple spectroscopy-based set-up for the simultaneous monitoring of three quality parameters of MWF and a mathematical model relating them to the most influential process factors relevant during use. For this purpose, the effects of MWF concentration, pH and nitrite concentration on the droplet size during aging were investigated using of a response surface modelling approach. Systematically varied model MWF fluids were characterized using simultaneous measurements of absorption coefficients µ_a_ and effective scattering coefficients µ’_s_. The droplet size was determined via dynamic light scattering (DLS) measurements. The droplet size showed non-linear dependence on MWF concentration and pH; however, the nitrite concentration had no significant effect. pH and MWF concentration showed a strong synergistic effect, which indicates that MWF aging is a rather complex process.

In the fourteenth, paper by Ni et al. [14], the authors demonstrated the applicability of random lasers as light sources and optical sensors. In a random laser (RL), optical feedback arises from multiple scattering instead of conventional mirrors. RLs generate a laser-like emission, while taking advantage of a simpler and more flexible laser configuration. Their applications have been extended to the biological field, with tissues used as natural scattering materials. Herein, the current state of the RL properties and applications was reviewed.

In the fifteenth paper, by Wang et al. [15], the authors present a review in order to give an overview of the historical development of waveguide-enhanced Raman spectroscopy (WERS) and highlight recent theoretical and experimental achievements with the technique. Photonic chip-based methods for spectroscopy are of considerable interest due to their applicability to compact, low-power devices for the detection of small molecules, and WERS has emerged over the past decade as a particularly interesting approach. WERS utilizes the evanescent field of a waveguide to generate Raman scattering from nearby analyte molecules, and then collects the scattered photons back into the waveguide. The large interacting area and strong electromagnetic field provided by the waveguide allow for significant enhancements in Raman signal over conventional approaches. The waveguide can also be coated with a molecular class-selective sorbent material to concentrate the analyte, thus further increasing the Raman signal.

In conclusion, the papers collected in this Special Issue summarize significant advances in the field of optical sensors, with specific contributions in sensor technology and applications of fiber Bragg gratings, resonators and spectroscopy.
